# Small-Scale Habitat Structure Modulates the Effects of No-Take Marine Reserves for Coral Reef Macroinvertebrates

**DOI:** 10.1371/journal.pone.0058998

**Published:** 2013-03-15

**Authors:** Pascal Dumas, Haizea Jimenez, Christophe Peignon, Laurent Wantiez, Mehdi Adjeroud

**Affiliations:** 1 Institut de Recherche pour le Développement (IRD), UR 227 CoReUs 2, Fisheries Department of Vanuatu, Port-Vila, Vanuatu; 2 Institut de Recherche pour le Développement (IRD), UR 227 CoReUs 2, Noumea, New Caledonia; 3 Smithsonian Environmental Research Center, Marine Invasions Research Laboratory, Tiburon, California, United States of America; 4 LIVE, Université de la Nouvelle-Calédonie, Noumea, New Caledonia; Universidade Federal do Rio de Janeiro, Brazil

## Abstract

No-take marine reserves are one of the oldest and most versatile tools used across the Pacific for the conservation of reef resources, in particular for invertebrates traditionally targeted by local fishers. Assessing their actual efficiency is still a challenge in complex ecosystems such as coral reefs, where reserve effects are likely to be obscured by high levels of environmental variability. The goal of this study was to investigate the potential interference of small-scale habitat structure on the efficiency of reserves. The spatial distribution of widely harvested macroinvertebrates was surveyed in a large set of protected *vs.* unprotected stations from eleven reefs located in New Caledonia. Abundance, density and individual size data were collected along random, small-scale (20×1 m) transects. Fine habitat typology was derived with a quantitative photographic method using 17 local habitat variables. Marine reserves substantially augmented the local density, size structure and biomass of the target species. Density of *Trochus niloticus* and *Tridacna maxima* doubled globally inside the reserve network; average size was greater by 10 to 20% for *T. niloticus*. We demonstrated that the apparent success of protection could be obscured by marked variations in population structure occurring over short distances, resulting from small-scale heterogeneity in the reef habitat. The efficiency of reserves appeared to be modulated by the availability of suitable habitats at the decimetric scale (“microhabitats”) for the considered sessile/low-mobile macroinvertebrate species. Incorporating microhabitat distribution could significantly enhance the efficiency of habitat surrogacy, a valuable approach in the case of conservation targets focusing on endangered or emblematic macroinvertebrate or relatively sedentary fish species

## Introduction

While coral reefs provide a wide array of environmental and economic services, concerns about their sustainability have dramatically increased over recent decades [1], [2]. Among the various threats resulting from the ever growing human impacts, resource depletion is a major issue for the ∼30 million people who are largely dependent on coral reefs for their livelihoods [3]. As a main consequence of overfishing, populations of many subsistence or commercial fish/invertebrate species are now seriously collapsing, creating local risks for food security throughout the Indo-Pacific [4].

In this context, there is an urgent need to promote relevant management solutions to reverse these alarming trends. It is now widely advocated that marine reserves (*sensu largo*, i.e. encompassing diverse management initiatives based on contrasted scales, closure regimes, target species, legislation etc.) constitute an effective restoration and conservation tool for commercial fish, whose benefits may in certain cases extend well beyond the physical boundaries of the protected area (e.g. [5]–[9] [10]). In contrast, little work has focused on invertebrates, especially in tropical areas. Despite their importance for the coastal fisheries of most Pacific insular countries, very few quantitative studies have investigated the ecological responses of traditionally harvested macroinvertebrates to protection [11].

On coral reefs, habitat structure may affect species' abundance and assemblages as well as their distribution through complex interactions between species' life histories and environmental factors [12], [13]. It is therefore a challenge to assess the actual effects of management measures, given its reliance on the ability to distinguish the direct influence of protection from the confounding effects of other sources of spatio-temporal variability [14], [15]. Macroinvertebrates usually exhibit a close linkage with the substrata that is derived from their life habits (feeding strategies, locomotory behavior, substrate relations etc.) [16]. Reserve effects with respect to life traits of the target species are thus likely to be obscured by variations in habitat structure occurring over a range of scales [17]. Together with relevant sampling of species/assemblages, addressing protection effects thus requires adapted (i.e. quantitative, low-bias) techniques to assess coral reef habitats at these scales.

The aim of this study was to investigate the influence of small-scale (10^1^ m) habitat structure and the related macroinvertebrate distribution on the effects of protective measures in coral reefs. In this study, widely harvested mollusk species (topshell *Trochus niloticus*, giant clams from the genus *Tridacna*) were surveyed in a large set of protected *vs.* unprotected stations across 11 reefs of the southwestern lagoon of New Caledonia. We sampled 250 transects of 20 m^2^ and 17 local habitat variables (sediment characteristics, reef structuring benthic species) in order to: 1) to investigate the relationships between the spatial distribution of species and their habitat structure at small scales; and 2) to elucidate the potential influence of habitat structure on the effectiveness of reserve implementation for coral reef invertebrates.

## Materials and Methods

### Study area

In New Caledonia, reef and lagoon formations together cover an area of approximately 22 200 km^2^, encompassing a significant level of biodiversity based on a large variety of benthic habitats [18]. All sampling sites were located on the shallow reefs of the southwestern lagoon of New Caledonia (22°170S and 166°300E). In the area, reefs are generally composed of three different consecutive zones in close proximity: a) a reef flat (depth range 0.5–2 m) with low coral cover; b) a reef crest (depth range 1–3 m) with flourishing corals; and c) a slight slope with coral cover similar to the reef crest and connecting the reef itself to the sandy bottom of the lagoon [19]. As a response to the increasing fishing pressure over recent decades, 18 marine protected areas (MPAs), including islets, reefs and bays, were implemented within the Southern Province of New Caledonia between 1981 and 2006. Most of them are located close to the city of Noumea, totaling 183 km^2^ over a total lagoon area of 4798 km^2^ [20].

**Figure 1:**
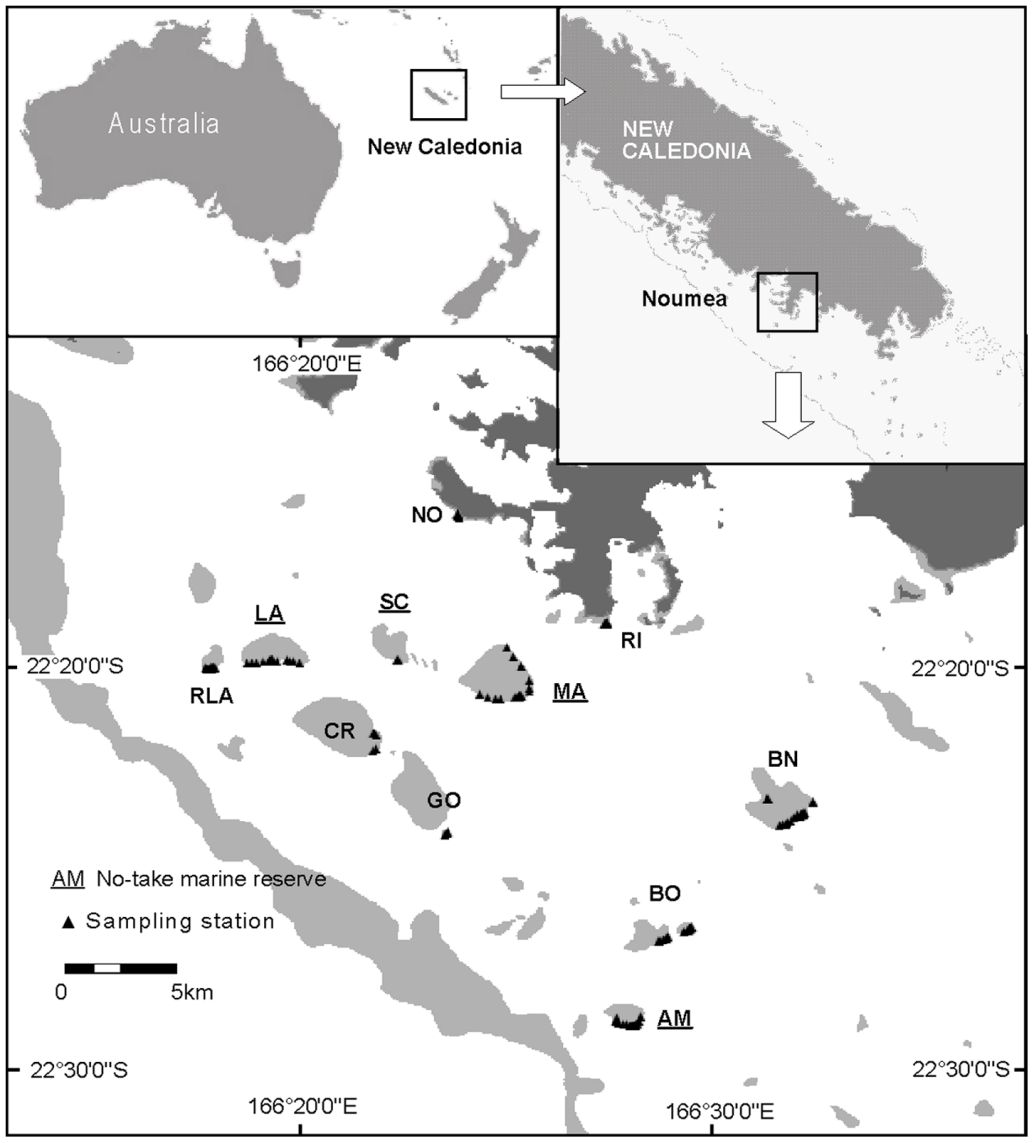
Study area. Location of the sampling stations, southwestern lagoon of New Caledonia (south Pacific).

In the area, sampling sites were preselected using geomorphological classification and available habitat maps so as to exhibit the environmental characteristics suitable for our target invertebrate species: hard-bottom habitats, essentially made up of rocky substrate (dead corals, boulders, debris) covered by sessile organisms (in particular live corals, algae, sponges) in zones subject to moderate to high wave action [21]. Eleven reef sites (four protected and seven unprotected reefs) exhibiting similar middle-scale habitat characteristics, depth range and exposure were finally retained for this study (Fig. 1). Permit to work inside the reserves was provided by provincial authorities (DENV, Direction de l'Environnement de la Province Sud, Noumea).

### Invertebrate sampling

The study focused on heavily exploited macroinvertebrate resources, i.e. giant clams (genus *Tridacna* and *Hippopus*) and trochus shells (*Trochus niloticus*). These large mollusk species have traditionally been consumed as seafood in the Pacific island countries for many centuries [22]. As a result of high local consumption and the growing demand for live specimens for the aquarium trade, all species of giant clams were listed in 1997 in Appendix B of the Convention on International Trade in Endangered Species of Wild Fauna and Flora (CITES). Similar concerns were raised for the mother-of-pearl shell *Trochus niloticus*, whose high value and non-perishable quality make it an increasingly attractive source of income for isolated island communities [23]. Despite the growing implementation of conservation measures, recent reports from international agencies indicate that trochus and giant clams are still seriously overfished across the whole Pacific, with a severe population collapse pushing these species to the brink of local extinction in many areas [24], [25].

The surveys were undertaken between 2007 and 2009 at the 11 reef sites. On each site and with respect to reef extension, up to 49 random transect belts of 20×1 m were laid using a color-marked survey tape attached to the substratum (Table 1). The depth range was 1–4 m; the distance between transects was a minimum of 5 m. The data were collected by two snorkelers swimming simultaneously along the transect line.

**Table 1:** Characteristics of the reef sampling sites, southwestern lagoon of New Caledonia (south Pacific).

Site	code	protection status	date	stations
Amédée reef	AM	protected	1981	39
Bancs du nord reef	BN	unprotected	-	23
Bancs de l'ouest reef	BO	unprotected	-	42
Crouy reef	CR	unprotected	-	5
Goêlands reef	GO	unprotected	-	5
Larégnère reef 1	LA	protected	1989	35
Larégnère reef 2	RLA	unprotected	-	29
Maitre reef	MA	protected	1981	49
Nouville reef	NO	unprotected	-	8
Ricaudy reef	RI	unprotected	-	10
Seche-croissant reef	SC	protected	1994	5

Reef code, status/date of protection and number of stations sampled.

All individuals belonging to the target species that were detectable along a 1 m-wide corridor without disturbing the substrate were identified to the species level and counted. Closely related, non-harvested trochus species (*Trochus maculatus, Tectus pyramis)* were also surveyed as “control species”. Individual sizes were recorded to the nearest 5 mm using calipers.

### Habitat sampling

For each transect, sediment type and substratum coverage variables were estimated using a recently developed photographic method to provide a quantitative, low-bias description of reef habitats [26]. Pictures were taken from the surface along all the transects using a digital 8 Mpixel Canon S80 camera in an underwater housing, oriented perpendicular to the substrate. Twenty pictures per transect (i.e. one shot every meter, providing a continuous photographic record of the transect) were recorded and subsequently imported into an image analysis program for the estimation of sediment/substrate cover (CPCe “Coral Point Count with Excel extensions” software) [27]. Seventeen local habitat variables were considered, related to sediment type and substratum coverage by large, sessile organisms and seagrass or turf+macroalgae (Table 2). Surface estimates expressed in percentage covers were derived from random stratified point count techniques using a 9 points.m^−2^ ratio in order to ensure reliable habitat profiles with a low bias and at high precision [26]. Percentage covers were then aggregated at the transect level.

**Table 2:** Habitat variables referring to sediment type, substratum coverage used for habitat characterization in the sampling sites, southwestern lagoon of New Caledonia (south Pacific).

Sediment type	Substrate coverage
Mud	Branching corals
Sand	Digitate corals
Rubble	Tabular corals
Boulders (<100 cm)	Massive corals
Dead Coral substrate	Submassive corals
Bedrock	Foliose corals
	Encrusting corals
	Soft corals (Alcyonarians)
	Fire corals (Milleporidae)
	Seagrass
	Turf+macroalgae

### Data analysis

The potential influence of sampling periods was tested using non-parametric Kruskal–Wallis ANOVAs on the density of target species. Interactions between closely related trochus species (*Trochus niloticus* vs. *Tectus pyramis*) with respect to protection status were investigated using two-factors PERMANOVA with subsequent pair-wise tests by permutation using 9999 permutations [28]. The influence of protection on the target species was first assessed globally using non-parametric Mann–Whitney U-tests on density/size data per species from protected *vs.* unprotected stations. The habitat-related effects of protection were then investigated using a combination of univariate and multivariate techniques. A multidimensional station similarity matrix based upon the calculation of Euclidian distances between the samples was built using the 17 substrate variables (sediment type, substratum coverage) [29]. Stations were then ordinated using non-metric multidimensional scaling (n-MDS) in order to establish a multifactorial typology of the habitat; groups (clusters) of transects sharing similar habitat were constituted using the similarity coefficients. Between-group discrimination was tested using PERMANOVA analyses performed on the 17 habitat variables, with subsequent pair-wise tests by permutation using 9999 permutations. Species abundances were then plotted on the resulting diagrams in order to determine visually species–habitat relationships. Density and size variations per species in relation to protection status were then tested for each habitat-derived group, using non-parametric Mann–Whitney and Kruskal–Wallis tests.

All analyses were performed using Statistica v.6 and Primer v.6 with PERMANOVA add-on statistical packages.

## Results

### Global effects of reserves on target species

In total, 3324 individuals belonging to the target species were counted and measured across the 250 reef stations. The results did not indicate significant variations in density across the sampling period for the harvested species. *Trochus* species were the most abundant and widespread, with 3044 individuals found at 219 stations (87.6%) across the study area. Giant clam species were less common, with 280 individuals belonging to three species (*Tridacna maxima, T. squamosa, Hippopus hippopus*) found at 105 out of the 250 sampled stations.

The density of harvested species was generally low, ranging from 0 to 2.1 individuals.m^−2^. The ecologically closely-related trochus species *Trochus niloticus* and *Tectus pyramis* had a similar range of density (mean 0.3 individuals.m^−2^) and were widely distributed across the study area (found in 76.8 and 75.2% of the sampled stations, respectively). Among giant clams, *Tridacna maxima* was clearly dominant (98.6% of the records, mean 0.05 individuals.m^−2^) but was present at only 25.6% of the stations. The other target species (*Trochus maculatus, Tridacna squamosa, Hippopus hippopus*) were found in very low abundance (≤1%) and were therefore not considered for further analyses. Size data encompassed a significant range of size classes from juveniles to adults, with shell diameters ranging between 2 and 14 cm/2 and 11 cm for *Trochus niloticus* and *Tectus pyramis*, respectively. In the area, *Trochus niloticus* usually exhibited larger sizes than *Tectus pyramis* (mean 9.1±2.3 cm/4.9±0.8 cm, respectively). The giant clam *T. maxima* exhibited sizes from 2 to 27 cm (mean 11.5±4.1 cm).

**Figure 2:**
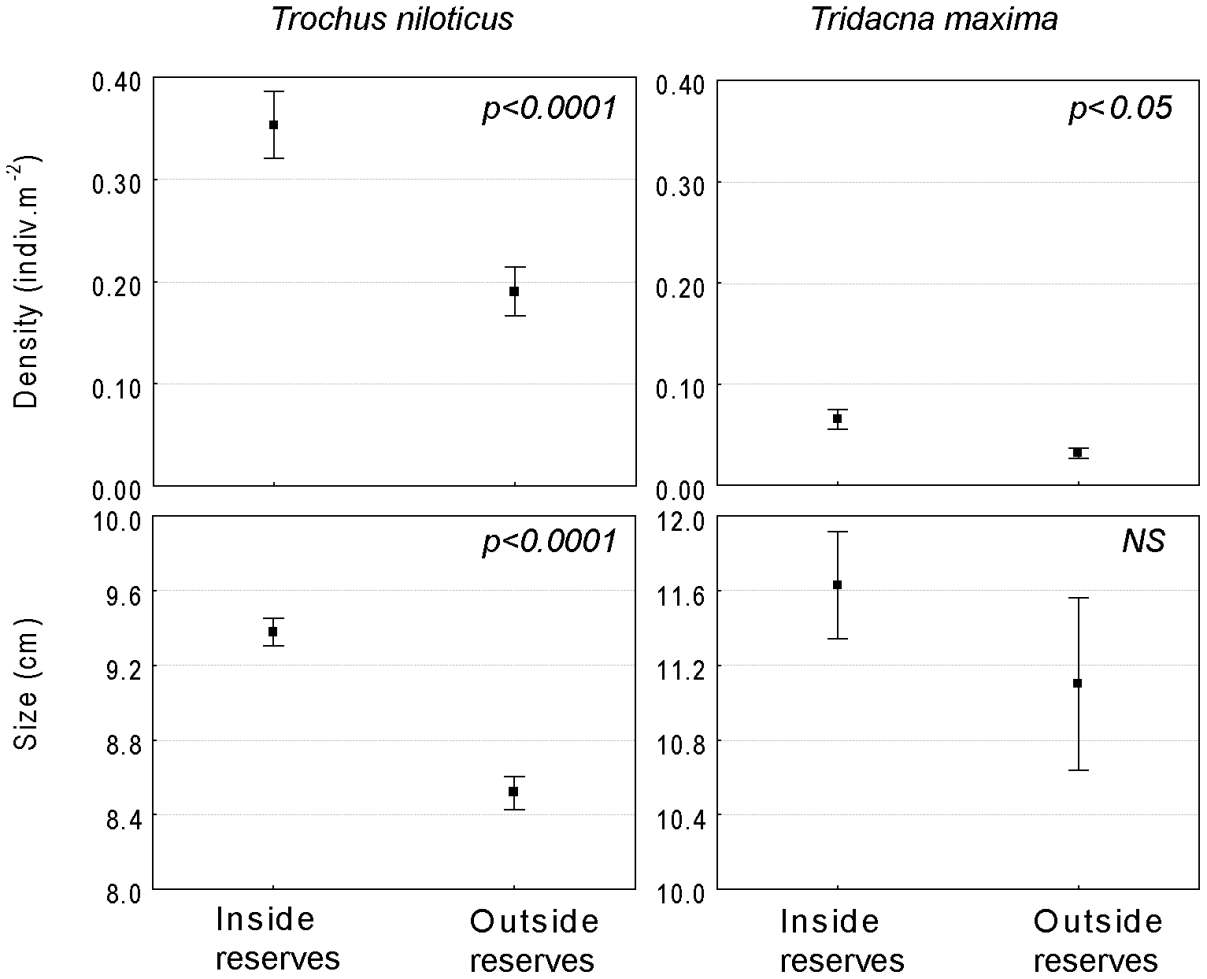
Effects of protection status for topshell *Trochus niloticus* and giant clam *Tridacna maxima*. Mean density (indiv.m^−2^) and size (cm) in protected *vs.* unprotected stations (mean±SE). Results of Mann-Whitney tests (^*^ p<0.05; ^**^ p<0.01; ^***^ p<0.001).

Irrespective of habitat, species-specific protection effects could be discerned for our target species (Fig. 2). Protected reef stations clearly exhibited larger populations of *T. niloticus*: mean density was nearly two times greater inside than outside the reserves (0.35 *vs.* 0.19 individuals.m^−2^, respectively; Mann–Whitney U-test, p<0.0001, n = 250). Reserves also harbored larger individuals (mean 9.4 cm inside *vs.* 8.5 cm outside reserves; Mann–Whitney U-test, p<0.0001, n = 1567). The same patterns were observed for giant clams, with their density increasing two-fold inside the reserves (0.06 individuals.m^−2^ inside *vs.* 0.03 individuals.m^−2^ outside, Mann–Whitney U-test, p<0.05, n = 250). There was a trend toward larger individuals inside the reserves, but the results were not statistically significant (mean 11.6 cm inside and 11.1 cm outside, Mann–Whitney U-test, n = 272, NS).

**Figure 3:**
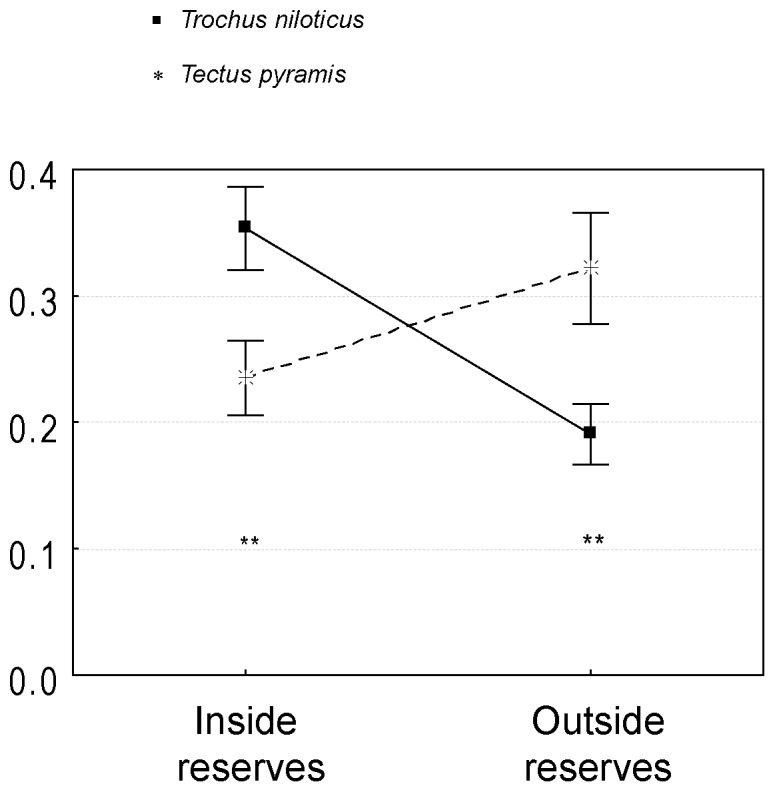
Interactions between two related trochus species. Mean density (indiv.m^−2^) of *Trochus niloticus* vs. *Tectus pyramis* in protected *vs.* unprotected stations (mean±SE). Results from Student tests (^*^ p<0.05; ^**^ p<0.01; ^***^ p<0.001).

Opposite density patterns were observed across the study area for the two major trochus species (Fig. 3). While *Trochus niloticus* was strongly dominant over *Tectus pyramis* in reserves, the opposite was true outside reserves (two-way PERMANOVA with subsequent pair-wise tests, pseudo-F = 14.05 for interaction species×protection status, p<0.001).

### Small-scale distribution of habitats

**Figure 4:**
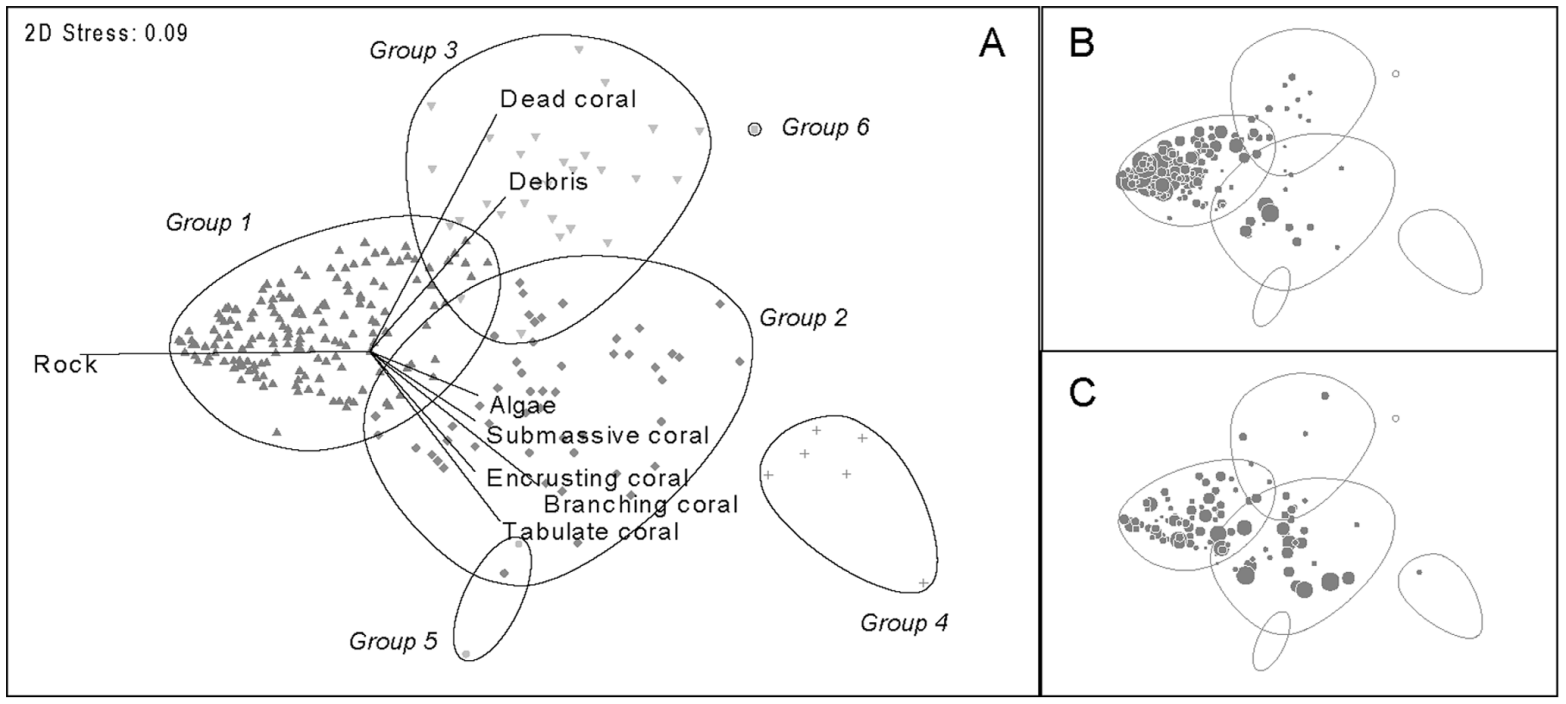
Two-dimensional MDS of the reef stations based upon habitat variables. **A.** Station plot showing the most determinant sediment type/substratum coverage variables (correlations >0.4). Same plots with grey circles proportional to the density of **B.** *Trochus niloticus* (adults) and **C.** *Tridacna maxima*.

In our 11 reef sites, the benthic habitat was mostly constituted of rocky substrate (mean cover 68.9±21.2%) with moderate living coral cover (22.7±18.8%) and low rubble (6.0±7.9%) or turf/macroalgae (1.6±5.5%). While stations were initially selected on the basis of similar geomorphological and medium-scale habitat features, analyses of the 17 substratum variables revealed distinct habitats at the transect (10^1^ m) scale. MDS plots highlighted six major, well-discriminated groups of reef stations delineating contrasting small-scale habitats (‘microhabitats’ hereafter) (PERMANOVA, Pseudo-F = 84.33, p<0.001; subsequent between-group pair-wise tests with p<0.001 for all pairs, Fig. 4A). Group H1 (left side of the diagram) encompassed 65% of the sampled transects. These stations were characterized by low structural complexity and a bedrock-dominated habitat with a low cover of coral (living or dead) and rubble. Group H2 (bottom of the diagram, 20% of sampled transects) captured stations with a higher living coral cover and a moderate to high structural complexity related to the presence of branching/tabular/sub-massive coral forms. Group H3 (top of the diagram, 11% of sampled transects) corresponded to detritic habitats mostly characterized by the presence of dead coral and rubble. Groups H4 to H6 were minor groups, with very low representation (3.6% of the sampled transects).

Plots of species density with respect to the latter groups suggested there were marked species-habitat relationships for both *T. niloticus* and *T. maxima* at this scale (Figs 4B, 4C). Station plots associated with significant trochus density were mostly found in reef microhabitat H1 (left side of the diagram). Stations with significant giant clam density were located in H2 and H3. At the lagoon scale, most reef sites exhibit mixed distributions of microhabitats, with stations mainly distributed over the three previously described major groups H1, H2 and H3. Only a few sites came under one single microhabitat (AM/GO reefs, 100% of stations in H1; NO/SC reefs, 100% of stations in H2).

### Reserve effects with respect to small-scale habitat distribution

**Figure 5:**
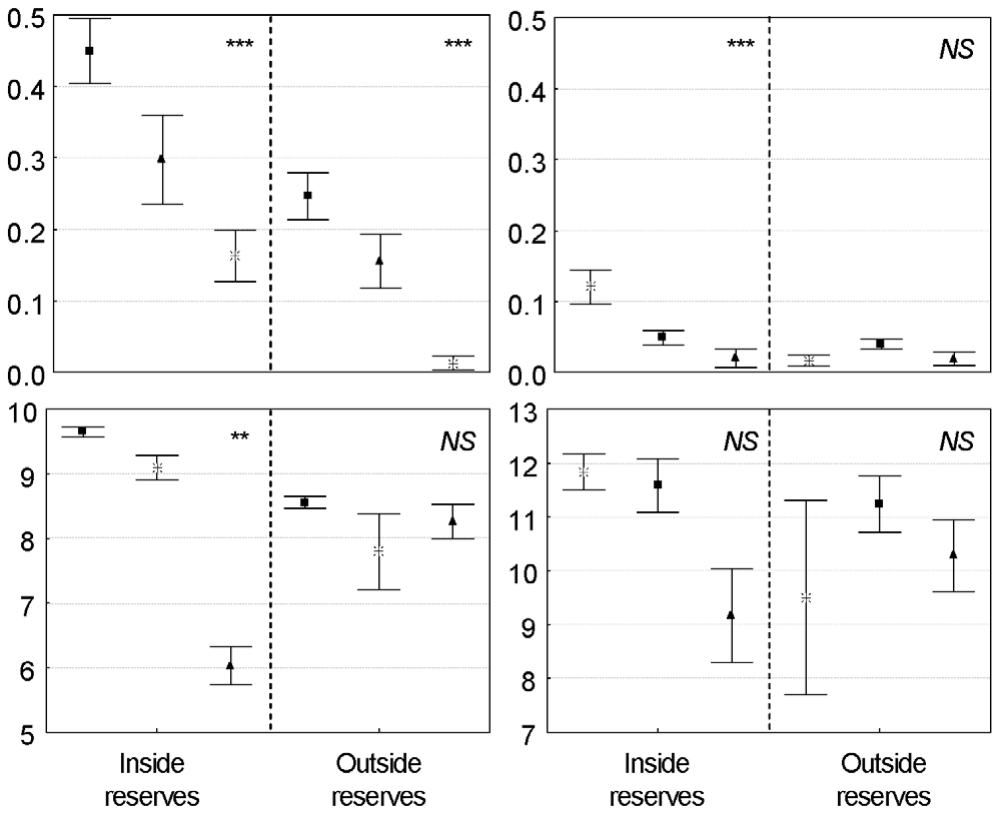
Influence of small-scale habitat on the distribution of target species. Mean density (indiv.m^−2^) and size (cm) plots for *Trochus niloticus* and *Tridacna maxima* in habitats H1, H2 and H3 with respect to protection status. Results of Kruskall-Wallis tests (^*^ p<0.05; ^**^ p<0.01; ^***^ p<0.001). ▪ habitat H1; ^*^ habitat H2; ▴habitat H3.

Testing reserve effects independently for H1, H2 and H3 provided a different picture, as the results highlighted that protection effects were not consistent across all habitats at this scale (Table 3). Very strong and positive responses were observed for *T. niloticus* in H1 (rock-dominated microhabitat), where both density and size increased markedly inside the reserves when compared to outside (Mann–Whitney tests, p<0.001 for both metrics). Protection effects were less marked in H2 and H3, as only partial effects could be detected in H2 (significant for density metric only, Mann–Whitney test, n = 50, p<0.01) and H3 (significant for size metric only, Mann–Whitney test, n = 119, p<0.001). In the reserves, microhabitat H1 always harbored larger populations of *T. niloticus*, with density nearly three times greater than in the coral-dominated H2 and 1.5 times greater than in the detritic H3 (0.45, 0.16 and 0.30 individuals.m^−2^ for reserve stations in habitats H1, H2, H3, respectively; Kruskal–Wallis test, p<0.001). The same pattern was even observed in unprotected stations, with H1 always exhibiting significantly higher densities compared to habitats H2 and H3 (0.25, 0.01 and 0.16 individuals.m^−2^ for the unprotected stations in H1, H2, H3, respectively; Kruskal–Wallis test, p<0.001) (Fig. 5).

**Table 3:** Reserve effect with respect to microhabitat in the sampling sites.

	All habitats		Habitat 1		Habitat 2		Habitat 3	
	*mean*	*p*	*mean*	*p*	*mean*	*p*	*mean*	*p*
Density								
*T. niloticus*	0.28	^***^	0.35	^***^	0.12	^***^	0.21	*NS*
*T. pyramis*	0.29	*NS*	0.35	*NS*	0.04	^*^	0.35	*NS*
*T. maxima*	0.05	^*^	0.04	*NS*	0.09	^**^	0.02	*NS*
Size								
*T. niloticus*	9.10	^***^	9.28	^***^	9.03	*NS*	7.15	^***^
*T. pyramis*	4.91	*NS*	4.95	*NS*	5.32	^*^	4.59	^***^
*T. maxima*	11.45	*NS*	11.43	*NS*	11.71	*NS*	9.77	*NS*

Mean density (indiv.m^−2^) and size (cm) for the target species in the considered habitats. Results of Mann-Whitney tests for AMP *vs.* non-AMP stations (^*^ p<0.05; ^**^ p<0.01; ^***^ p<0.001).

In the same way, while only moderate reserve effects were initially detected across the area for the giant clam *T. maxima*, the results revealed marked differences when testing protection effects for each microhabitat independently. Strong, significant differences were found in coral-dominated microhabitat H2, where the mean density increased by up to six-fold between protected *vs.* unprotected stations (0.12 and 0.02 individuals.m^−2^ for protected and unprotected stations, respectively; Mann–Whitney U-test, n = 50, p<0.01). No differences were detected in the other microhabitats.

## Discussion

### Reserve efficiency for coral reef invertebrates: general trends

Despite the existence of a large amount of grey literature, including reports from government agencies, NGOs and local communities, there is limited quantification of the efficiency of reserves for traditionally harvested macroinvertebrates in the Pacific.

Our findings demonstrate that marine reserves can substantially augment the local density, size structure and biomass of the heavily-exploited trochus and giant clam species. Effect magnitude appeared to be mostly reserve (i.e. site)-specific: while density doubled globally for both trochus shells and giant clams at the lagoon scale, it eventually reached levels four times greater in some particular reserves. The average size was raised by 10 to 20% for trochus species, depending on the site, but not for giant clams. Interestingly, our results highlighted markedly opposite density patterns for *T. niloticus* and non-harvested *Tectus pyramis,* suggesting competitive interactions between these two closely-related species, which grow to almost the same size and share very similar ecological niches. The same patterns were observed by Lincoln-Smith [30], who hypothesized a limitation of the abundance of *T. pyramis* caused by competition with *T. niloticus* inside the reserves, while depletion of *T. niloticus* in unprotected sites allowed abundances of *T. pyramis* to increase.

On the whole, our findings were strikingly consistent with the conclusions raised by the meta-analysis of Halpern [31], which found that creating a reserve appeared to double the density and increase organism size by 20–30% on average, even though the data from the 89 studies used for the meta-analysis were synthesized across vastly different ecosystems (including non-reef and temperate marine systems) and covered mostly fish species. In coral reefs, substantial benefits of protection were reported for some heavily-harvested invertebrate species including mollusks, crustaceans and holothurians [32]–[35]. Reserve benefits ultimately depend on interacting factors such as reserve features (e.g. size, location, restrictions, and enforcement), local environmental conditions (including the availability of suitable habitat for juveniles and adults) and the biology/ecology of the target species [36]. A rapid, significant increase in density and mean size of organisms may be expected for fast-growing species such as *Trochus niloticus*, as long as the threshold density of adult conspecifics that ensures natural recovery is maintained [37]. In the Solomon Islands, a three-fold increase in the density of trochus shells was reported after only three years of protection [30]. A similar response was recently documented in Vanuatu, where trochus density was three times greater inside a village-based reserve after four years of protection [38]. Yet, in the latter case, the synergistic effects of protection and punctual translocation of adult trochus individuals inside the reserve by local fishers may enhance reserve outcomes. On the other hand, decades may be required before some of the positive effects eventually become visible for severely depleted, vulnerable species exhibiting low, erratic reproductive success, such as giant clams, holothurians or large gastropods [39]–[42].

### Spatial variability of “reserve effect” in reef systems

While the efficiency of the New Caledonian reserve network was incontestable at the global (lagoon) scale, an inconsistency in invertebrate responses arose at the smaller (reef) scale. Transects with a higher trochus/clam density and/or sizes were punctually observed outside the reserves, highlighting localized, unexpectedly high invertebrate “hotspots”. Conversely, some transects with surprisingly low invertebrate values were scattered inside the reserves apparently at random, with size/density well below the average values. As a consequence, the apparent efficiency of reserves in restoring/enhancing macroinvertebrate populations appeared to be strongly dependent on the spatial scale (transect, reef, lagoon) at which it was investigated.

This ecological “background noise” is a well-known consequence of the multiple sources of variability occurring in natural ecosystems. In particular, a marked spatial heterogeneity at various scales appears to be a distinctive characteristic of coral reef communities [43], [44]. Depending on the taxa considered and the local environmental conditions, heterogeneity may be realized at various scales within a reef system: from small-scale (i.e. sampling station, typically 10^0^–10^2^ m, or lower) to meso-scale (geomorphological units and reefscape, 10^1^–10^2^ m) and large-scale (whole reefs, 10^2^ m and above). In contrast with most non-sedentary fish species whose variability is typically described over medium to large scales (10^2^–10^5^ m; see review in [45]), the small-scale heterogeneity of the physical substrate constitutes a major structuring factor for tropical macroinvertebrates. This is in particular expected for sessile or large, sedentary species such as trochus shells and giant clams, whose distribution may be better captured at small (metric) scales [46], [47]. Yet, most environmental or conservation-oriented studies tend to focus on more traditional, GCRMN-derived larger sampling areas (≥100 m^2^; [48]), making it difficult to discern the potential interference of habitat structure at smaller scales.

### Synergistic effects of protection and microhabitat distribution

Within apparently homogeneous geomorphologic reef units, our results thus emphasized the presence of contrasting microhabitats that differed in their relative proportions of bare substrate, living coral (in particular structurally complex branching and tabular morphotypes) and rubble cover. In the eleven sites studied, the reef substrate was identified as a mixture of three major microhabitats, emphasizing marked, within-reef spatial heterogeneity and patchiness. Of course, microhabitat patchiness may be further enhanced by marked variation in other small-scale biotic/abiotic factors that were not specifically measured in this study, e.g. including water chemistry, hydrodynamics or microbial diversity [49], [50]. Whatever the location or protection status, a strongly contrasted population density and, to a lesser extent, organism size were found with respect to the latter microhabitats, suggesting that the sampling scale considered was ecologically relevant for the target macroinvertebrate species. For adult trochus, distribution patterns are probably the consequences of marked ecological preferences linked to the particular locomotion and/or feeding behavior of this large, crawling species [51]. In the same way, while *Tridacna maxima* was more ubiquitous at the reef scale, as suggested by Dumas [52], it was preferentially observed in all reef areas except for patches of substrate exhibiting substantial rubble cover.

On the whole, this study highlighted the necessity of using habitat scales relevant to the ecology of the species considered in framing any ecological assessment of reserve efficiency. In New Caledonia, beyond the influence of landscape parameters operating at the large scale (e.g. geomorphology, hydrodynamics), the efficiency of reserves thus appeared to be locally modulated by the availability of suitable microhabitats at decimetric scales for the sessile/low-mobility macroinvertebrate species considered. Similar conclusions can be drawn from recent invertebrate surveys elsewhere in New Caledonia, with strong, habitat-specific protection effects usually observed for *Trochus niloticus* species at small, intra-reef spatial scales [53], [54].

### Perspectives: encompassing microhabitats to enhance the management of macroinvertebrate resources

**Figure 6:**
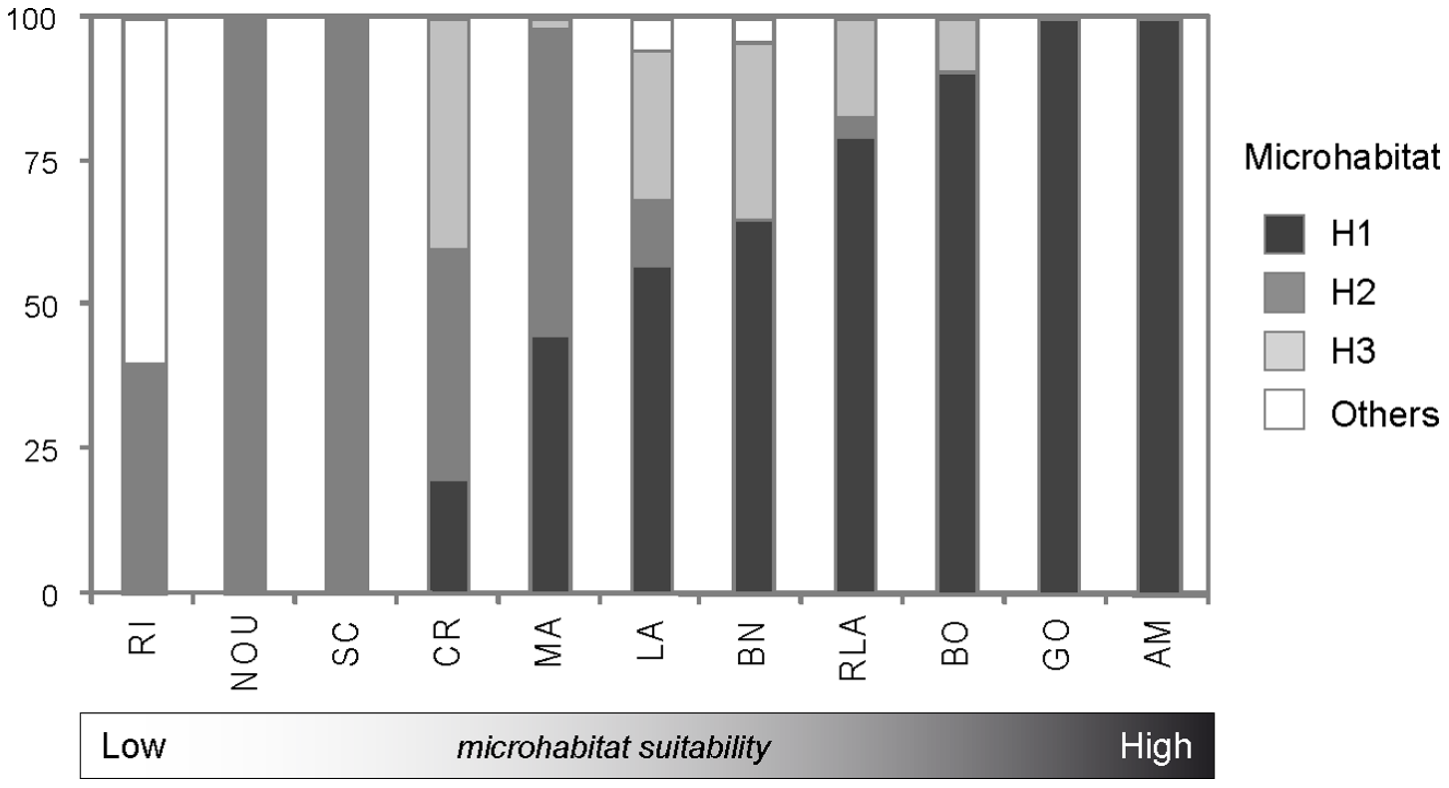
Potential for enhancement of *Trochus niloticus* populations within the reserve network of the southwestern lagoon of New Caledonia. Ranking of the 11 reef stations based upon microhabitat distribution. H1, H2, H3, others: microhabitat with decreasing suitability for *T. niloticus*. Reserves are figured with black squares.

Our results therefore clearly demonstrate that the apparent success (or failure) of reserves can be obscured by marked variations in population structure occurring over very short distances, resulting from small-scale patchiness of the coral reef habitat. Close, geomorphologically similar reefs are likely to exhibit a highly contrasted potential for invertebrate restoration and/or enhancement. This was evidenced in our reserve network, where all the reserves but one (Amédée reef) harbored a significant proportion of ‘non-optimal’ microhabitats for our target species (from 43 to 100%; Fig. 6). Conversely, unprotected reefs eventually exhibited better cover of suitable microhabitat than did reserves, suggesting higher ecological potential for the restoration of macroinvertebrate populations. Yet, this may vary through time, in particular under the influence of catastrophic events such as cyclones, which are likely to increase rubble cover, hence redistributing the proportion of bare substrate/coral-dominated/rubble microhabitats at the reef scale [55]. Increasing anthropogenic disturbance in the Noumea lagoon may also alter coral-algae competitive interactions, which plays a major role in structuring benthic coral reef communities and habitat [56]. Turf/macroalgae cover was generally very low in all the reef sites and did not constitute a major structuring variable in discriminating the microhabitats. In the light of previous monitoring surveys, our results do not currently support the hypothesis of phase-shift from coral to algal dominated microhabitats in the studied area, at least in the present phase [57].

Based on our results, encompassing the distribution of microhabitats strongly enhances the accuracy of macroinvertebrate mapping, which makes particular sense in the context of the spatially-explicit approaches increasingly being promoted for the management of coral reef resources [58], [59]. Decisions on the design and location of most reserves have largely been the result of tradeoffs between political/social processes and biological/environmental considerations [60]. There is now a growing consensus towards more holistic, ecosystem-based approaches using habitat maps as surrogates of biological information [61]–[63]. Considerable progress has been made during the last decade in the mapping of reef habitats at increasingly larger spatial scales, in particular given the now routine availability of high-resolution satellite imagery [64]. Yet, despite active research in ecological modeling, the scaling and mapping of biological resources in coral reefs remains a challenging task [65]. This is especially true for sedentary invertebrates, which may exhibit low congruence with habitat maps derived at non-ecologically relevant spatial scales [66]. With current trends moving towards more integrated approaches, incorporating microhabitat distribution derived from fine-scale habitat mapping could significantly enhance the efficiency of habitat surrogacy, a valuable approach in the case of conservation targets focusing on endangered or emblematic macroinvertebrate or relatively sedentary fish species.
